# Development of the FAST-DOSE assay system for high-throughput biodosimetry and radiation triage

**DOI:** 10.1038/s41598-020-69460-7

**Published:** 2020-07-29

**Authors:** Qi Wang, Younghyun Lee, Igor Shuryak, Monica Pujol Canadell, Maria Taveras, Jay R. Perrier, Bezalel A. Bacon, Matthew A. Rodrigues, Richard Kowalski, Christopher Capaccio, David J. Brenner, Helen C. Turner

**Affiliations:** 1 Center for Radiological Research, Columbia University Irving Medical Center, New York, NY 10032 USA; 2 Radiation Oncology, Columbia University Irving Medical Center, New York, NY 10032 USA; 3 ASELL, LLC, Owings Mills, MD 21117 USA; 4 Luminex Corporation, Seattle, WA 98119 USA

**Keywords:** High-throughput screening, Imaging, Biological techniques, Biomarkers

## Abstract

Following a large-scale radiological incident, there is a need for FDA-approved biodosimetry devices and biomarkers with the ability to rapidly determine past radiation exposure with sufficient accuracy for early population triage and medical management. Towards this goal, we have developed FAST-DOSE (**F**luorescent** A**utomated** S**creening** T**ool for** Dos**im**e**try), an immunofluorescent, biomarker-based system designed to reconstruct absorbed radiation dose in peripheral blood samples collected from potentially exposed individuals. The objective of this study was to examine the performance of the FAST-DOSE assay system to quantify intracellular protein changes in blood leukocytes for early biodosimetry triage from humanized NOD-scid-gamma (Hu-NSG) mice and non-human primates (NHPs) exposed to ionizing radiation up to 8 days after radiation exposure. In the Hu-NSG mice studies, the FAST-DOSE biomarker panel was able to generate delivered dose estimates at days 1, 2 and 3 post exposure, whereas in the NHP studies, the biomarker panel was able to successfully classify samples by dose categories below or above 2 Gy up to 8 days after total body exposure. These results suggest that the FAST-DOSE bioassay has large potential as a useful diagnostic tool for rapid and reliable screening of potentially exposed individuals to aid early triage decisions within the first week post-exposure.

## Introduction

In the event of a large-scale radiological incident or accident, hundreds of thousands of people may be exposed to ionizing radiation. There is an important need for the development of FDA-approved point-of-care radiation biodosimeters and in vitro diagnostic devices (IVDs) with the ability to rapidly determine past radiation exposure with sufficient accuracy for early population triage and medical management. Biodosimetry technologies may be designed for early in-the-field triage (usually qualitative) or for more clinical evaluation and medical management that includes dose level confirmation (usually quantitative or semi-quantitative)^1^. We have recently developed a new high-throughput biodosimetry assay system called FAST-DOSE (Fluorescent Automated Screening Tool for Dosimetry) which is designed for rapid immune-detection and quantitation of radiation responsive protein biomarkers and reconstruction of dose in human peripheral blood leukocytes. This biomarker-based triage assay has large potential as a useful diagnostic tool for the mass-screening of potentially exposed individuals and offers a short “time-to-result” since cell culture is not required compared with the gold standard micronucleus and dicentric biodosimetry assays^2–4^.

The FAST-DOSE assay device is based on imaging flow cytometry (IFC)^5–7^ and a panel of intracellular biomarkers identified by earlier proteomic study^8^ to rapidly quantify the upregulation of biomarker expression in blood leukocytes using fluorescent imagery and algorithms for the estimation of absorbed dose. The advantage of IFC (ImageStream, Luminex, Austin, TX) technology is that it combines the speed and quantification power of flow cytometry with the imaging capability of a conventional microscope^9^. The IFC simultaneously captures fluorescent and brightfield (BF) images at rates of more than 1,000 cells per second that enables several different structures within the cell to be analyzed in tens of thousands of cells in just a few minutes^7^. All BF and fluorescent images captured from IFC are stored in sample specific data files that allows analysis to be performed at any time post-acquisition using software such as the Image Data Exploration and Analysis Software (IDEAS). Within IDEAS, all dots on a scatter plot can be selected to display all corresponding imagery, which permits the creation of hand-selected ground truth populations based on visual confirmation of images containing desired features. This in turn, allows for the development of, efficient and informed gating strategies which can easily be applied to many data files using the batch processing option within the software. Furthermore, desired statistics such as the total number of gated events, intensity of fluorescence as well as quantitative measurements of morphological features (e.g. area, circularity, staining homogeneity, etc.) on a cell-by-cell basis, can be generated and exported^10^.

The objective of this study was to validate a panel of leukocyte protein biomarkers (ACTN1, BAX, DDB2, FDXR, phospho-p53 (p53) and TSPYL2) identified in our earlier work^8^ to reconstruct absorbed dose in peripheral blood in vivo using humanized NOD scid gamma (Hu-NSG) mice and non-human primates (NHPs) after total body irradiation (TBI) exposure. The candidate biomarkers are known to be associated with radiation-induced apoptosis, DNA damage and cellular senescence. Briefly, ACTN1 is an actin binding protein regulating actin cytoskeleton, and is one of the cellular senescence related proteins^11,12^. BAX is a well-known regulator in radiation-induced apoptosis through ATM and CHK2 mediated p53 activation^13^. DDB2 is a protein associated with nucleotide excision repair and has a key role in DNA damage recognition^14^. FDXR is a mitochondrial flavoprotein transferring electron from NADPH to cytochrome P450 which can be induced by DNA damage and is involved in p53 and oxidative stress-mediated apoptosis^11,15^. TSPYL2 is important for G1 checkpoint maintenance upon DNA damage^16^. In this work, we used IFC technology to rapidly quantify changes in these intracellular biomarker expression levels using a small amount of blood for high-throughput screening.

Hematopoietically humanized mice represent an alternative model to study the in vivo human biological response after ionizing radiation exposure. Since the description of immunodeficient mice bearing mutations in the IL2 receptor common gamma chain (*IL2rg*^null^) in the early 2000s, investigators have been able to engraft murine recipients with human hematopoietic stem cells (HSCs) that develop into functional human immune systems^17^. Hu-NSG mice may be generated following the transplantation of HSCs derived, for example, from bone marrow^18^, umbilical cord blood^19^, fetal liver^20^ or mobilized peripheral blood^20^ into NOD-*scid IL2rg*^*null*^ mice (NOG or NSG) mice or other strains, leading to the development of humanized hematopoietic progenitor and differentiated cells in the mouse bone marrow, spleen and thymus^20–22^. Recently, we have used the Hu-NSG model for radiation studies to support the development of biodosimeters to estimate absorbed dose in human blood leukocytes^8,23^.

The NHP model is considered the gold standard animal model for drug development^24^ whereby the NHP provides > 93% DNA sequence homology with humans^25^, and a high level of similarity in terms of response to physiological pathways and cell receptors^26,27^. The NHP model has also been used to study radiation response and injury^25^ providing important information about the dose response relationships and mitigation of hematological effects^28,29^, injury to the lung^30^ and gastro-intestinal system^31^ after ionizing radiation exposure. Recent studies have also shown persistent nuclear damage and gene expression changes in peripheral blood samples after exposure to 10 Gy ionizing radiation to the NHP whole thorax^27^.

Presented here, the Hu-NSG mice studies were designed to measure FAST-DOSE biomarker expression levels in human blood leukocytes at multiple early time points (days 1, 2 and 3) after acute-dose (0, 1 and 3 Gy) radiation exposure whereas for the NHP study, the dose range was expanded to 10 Gy TBI (0–6, 8 and 10 Gy) and biomarker levels were measured at specific time points up to 8 days (days 2, 4 and 8) post-exposure. Dose estimation algorithms were developed using univariate or multivariate linear regression analysis based on individual biomarker levels and their combination was used to estimate absorbed radiation dose in blood leukocytes. The FAST-DOSE assay biomarkers were able to generate delivered dose estimates within ± 0.04–0.61 Gy, at days 1, 2 and 3 after exposure in humanized mice, whereas in the NHP blood samples from fewer animals, the biomarkers were able to successfully classify samples by dose categories below or above 2 Gy.

## Results

### Reconstitution of human hematopoietic cells in humanized mice

**Figure 1:**
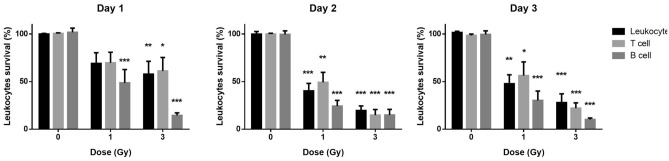
Human leukocyte cell survival after radiation exposure. The surviving percentage of human CD45+ leukocytes, CD3+ T and CD19+ B cells in the post-irradiated humanized mouse blood relative to the pre-irradiation is shown. Bars represent the mean surviving fraction of each group while the error bars represent the standard error of mean (SEM) (*p < 0.05; **p < 0.01 and ***p < 0.001).

Recipient NSG mice showed successful engraftment 3 months after injection of human fetal liver stem cells (CD34+ cells). Forty-six generated humanized mice had 61.1 ± 19.3% human cells (CD45 +), mostly human B and T cells. Figure 1 shows the percentage of depletion of human cells across the three dose groups (0, 1 and 3 Gy) on days 1, 2 and 3 post-exposures. Prior to irradiation, each group showed a similar proportion of human leukocytes, B and T cells (*p* > 0.05).

Figure 1 shows that in general, exposure to X rays decreased human leukocytes (*p* = 0.014). On day 1 post-irradiation, human B cells were significantly decreased at 1 Gy (*p* < 0.001), while human leukocytes, T cells and B cells were all significantly decreased at 3 Gy (*p* = 0.029 and *p* < 0.001, respectively). On days 2 and 3 post-irradiation, human leukocytes, T cells and B cells were all significantly decreased after 1 Gy (Day 2: T cell: *p* = 0.005, B cell: *p* < 0.001; Day 3: T cell: *p* = 0.041, B cell: *p* < 0.001) and 3 Gy (Day 2: T cell: *p* < 0.001; B cell: *p* < 0.001; Day 3: T cell: *p* < 0.001; B cell: *p* < 0.001). When leukocyte cell survival is compared between the two doses (1 Gy vs. 3 Gy) across different days, the results indicate that on day 1, B cells showed a dose dependent decrease (1 Gy vs. 3 Gy: *p* = 0.038, statistical significance not marked on the figure), while on days 2 and 3, T cells showed dose dependent decrease (Day 2: 1 Gy vs. 3 Gy: *p* = 0.017, Day 3: 1 Gy vs. 3 Gy: *p* = 0.039, statistical significance not marked on the figure).

### Quantification of biomarker expression using IFC in human leukocytes in vivo

**Figure 2:**
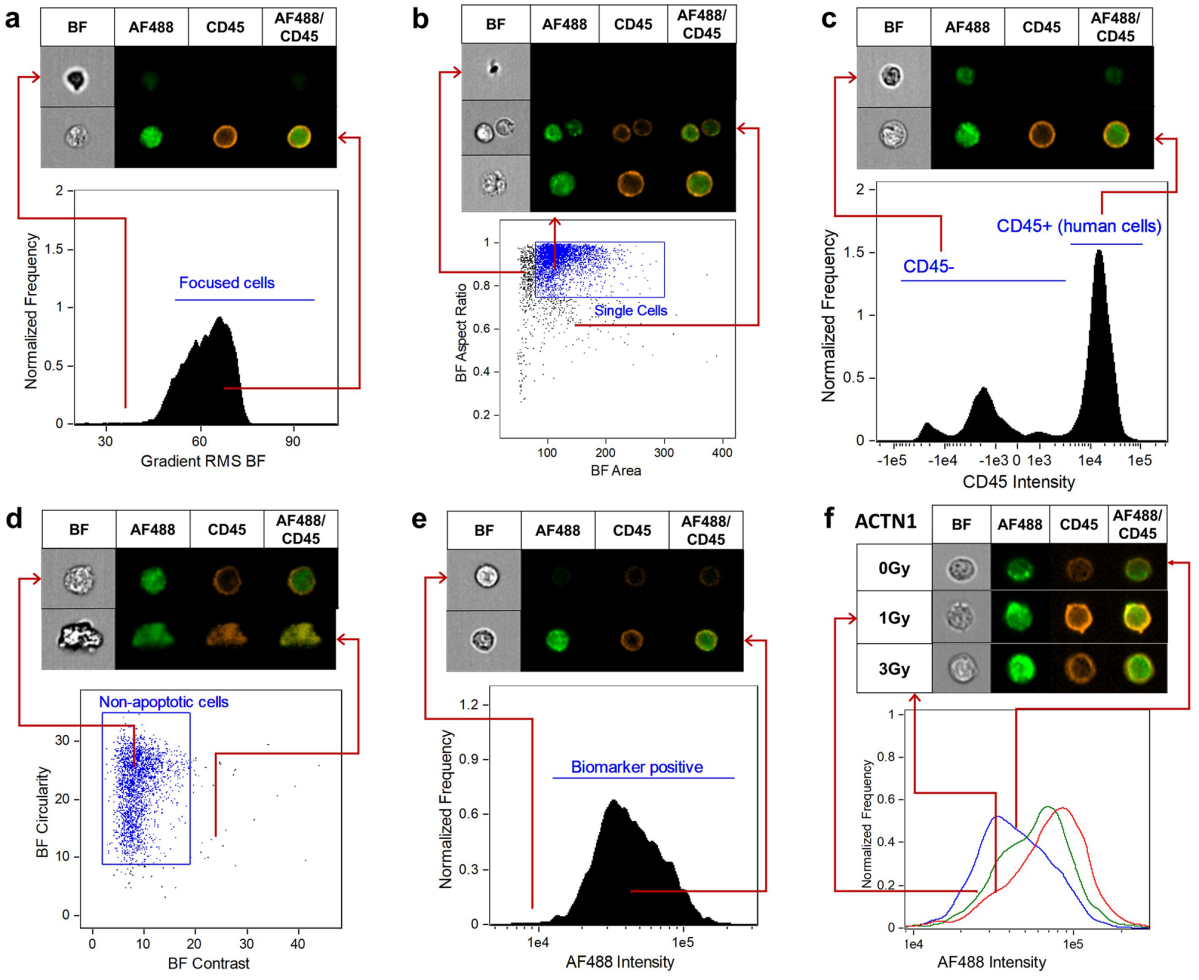
Representative analysis template in the IDEAS software. (**a**) The Gradient Root Mean Squared (RMS) feature was used to identify focused cells in the brightfield (BF) channel and to eliminate blurred images; (**b**) A bivariate plot of BF Area versus BF Aspect Ratio permits gating single cells and removing doublets or large debris; (**c**) Human leukocytes were then selected by gating on CD45 positive cells; (**d**) non-apoptotic cells were gated through the use of a bivariate plot of BF circularity versus BF contrast. Cells with low circularity and high contrast are apoptotic events and can be easily eliminated; (**e**) histogram of Alexa Fluor 488 intensity for quantifying mean fluorescence intensity of biomarkers; (**f**) representative biomarker expression and images of ACTN1 pre-and post- X-ray irradiation (1 Gy and 3 Gy).

**Figure 3:**
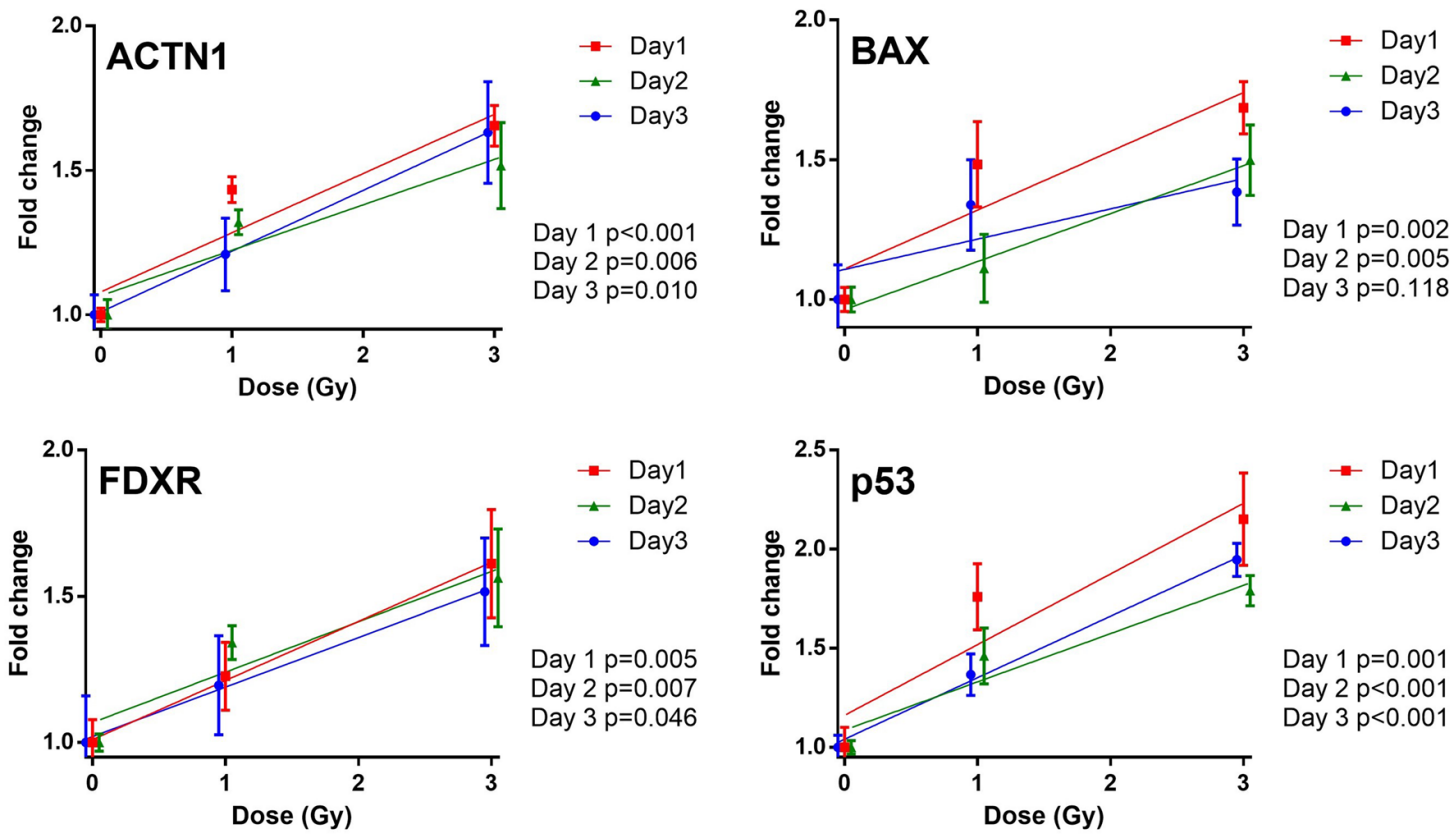
Radiation-induced changes in biomarker expression in CD45 positive human leukocytes from humanized mice on days 1, 2 and 3 post-irradiation. The results demonstrate a dose response relationship in the MFI for all biomarkers (ACTN1, BAX, FDXR and p53). Dose response curves from each day are shown (red: Day 1, green: Day 2; blue: day 3). The error bars represent the standard error of mean (SEM) and *p* values reflect the significance for linear regression.

Radiation induced protein expression levels were evaluated in human CD45+ cells in vivo from the peripheral blood of the humanized mice using IFC, with data analysis being performed using IDEAS software. The Gradient RMS feature was used to gate on focused cells in the BF channel, permitting eliminations of blurred events. The region boundary was set by visual inspection of cell images in the bright field channel (Fig. 2a). Single cells were then selected using a BF Area versus BF Aspect Ratio bivariate plot (Fig. 2b) and human leukocytes were selected by gating on CD45+ cells (Fig. 2c). As apoptotic cells may show a different morphological pattern in BF imagery^32^, they were then excluded using a BF Circularity versus BF Contrast plot (Fig. 2d). The mean fluorescence intensity (MFI) of each biomarker within the single cell population was then computed and exported from the IDEAS software (Fig. 2e). Representative images (Fig. 2f) of biomarker expression (ACTN1) observed in human CD45+ leukocytes after exposure to X rays (1 Gy and 3 Gy) are shown. We developed a uniform and simplified analysis template to quantify the MFI each biomarker in non-apoptotic CD45+ human leukocytes. This template was then applied to all data files and automatically batch processed within IDEAS. Figure 3 shows the dose response curves for ACTN1, BAX, FDXR and p53 expression from a total of 46 humanized mice, up to 3 days post-irradiation. Biomarker intensity for non-irradiated human leukocytes as well as mice irradiated with 1 Gy and 3 Gy X rays show that fluorescence intensity fold change increased with dose and is the highest following 3 Gy irradiation, as expected (Fig. 3). Associations of biomarker expression and irradiated dose were examined by linear regression, and all biomarkers displayed a dose-dependent response to radiation (*p* < 0.05; *p* values are shown in Fig. 3). To assess the diagnostic ability of the biomarkers for high and low dose in Hu-NSG mice, ROC curve analysis was performed to discriminate low doses (0 and 1 Gy) vs. high dose (3 Gy) (Supplementary Table S1). The results show that all biomarkers individually, were able to discriminate these two groups with AUCs ranging from 0.803 to 0.985, except for BAX at day 3.

### Dose reconstruction in humanized mice

Four biomarkers ACTN1, BAX, FDXR, p53 and their combinations were tested by linear regression to reconstruct the delivered dose. Each model was ranked according to information-theoretic support from the data, based on Akaike information criterion (AICc) scores with sample size correction. Since increasing the number of combined biomarkers in a particular model will bring an overwhelming penalty for best-fit predictor of dose in a small sample size cohort, only one or the combination of two biomarkers was selected for this analysis (Table 1). The results show that ACTN1 at day 1, p53 + BAX at day 2 and p53 at day 3 had the lowest AICc and were selected as best-fit predictor for dose with an accuracy of ± 0.04–0.61 Gy (Table 1 and Supplementary Figure S1). The ability of these multi-protein models to estimate dose including the mean absolute errors and coefficients of determination is shown in Table 1. The results demonstrate that although the combination of four biomarkers does not significantly improve dose estimation due to the penalty of the multiple predictors, they still have a strong association with dose and provide a valuable estimation (Table 1 and Supplementary Table S2).

**Table 1:** Dose estimation ability in humanized mouse model.

	Model	Estimated dose (mean ± SEM, Gy)			R^2^ adjusted (*p* value)	MAE	AICc
		Delivered dose					
		0 Gy	1 Gy	3 Gy			
Top ranking biomarker	Day 1—ACTN1	− 0.04 ± 0.08	1.61 ± 0.13	2.51 ± 0.20	0.75 (< 0.001)	0.46	− 10.35
	Day 2—P53 + BAX	0.19 ± 0.33	1.30 ± 0.35	2.54 ± 0.49	0.71 (< 0.001)	0.46	− 6.03
	Day 3—P53	0.21 ± 0.16	0.93 ± 0.19	2.65 ± 0.24	0.78 (< 0.001)	0.45	− 10.93
Combination of 4 biomarkers	Day 1	− 0.09 ± 0.10	1.58 ± 0.15	2.44 ± 0.27	0.73 (< 0.001)	0.45	− 4.50
	Day 2	0.18 ± 0.33	1.31 ± 0.35	2.54 ± 0.50	0.64 (< 0.001)	0.48	1.03
	Day 3	0.17 ± 0.17	1.02 ± 0.16	2.67 ± 0.29	0.75 (< 0.001)	0.43	− 3.27

AICc, R^2^ and *p* values were obtained by linear regression analysis for estimated versus delivered doses. SEM: standard error of the mean. MAE: mean absolute error, values were used as indicators to compare the difference between delivered and estimated dose.

### Biomarker performance in the NHP model

**Figure 4:**
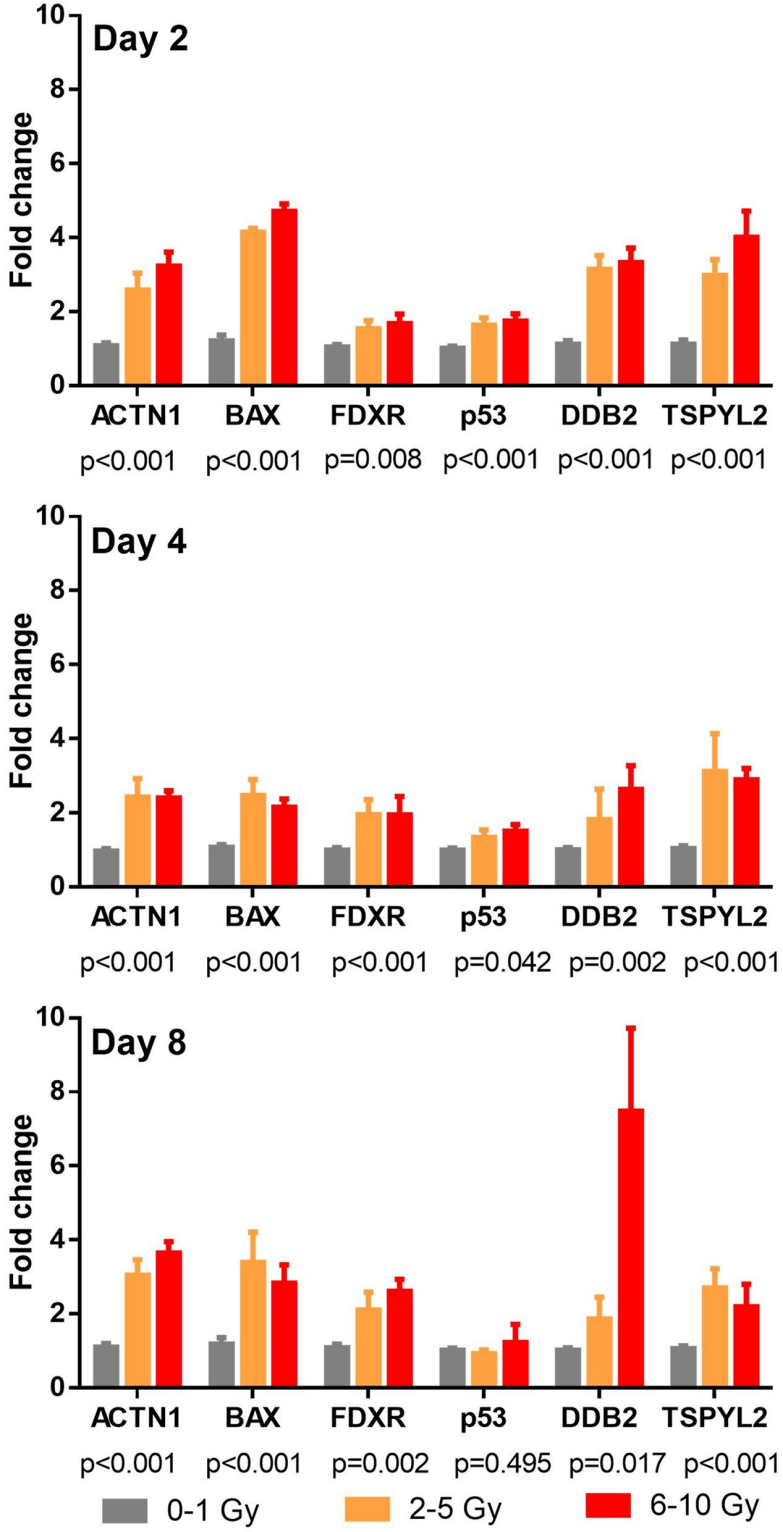
Fold change of biomarker expression ACTN1, BAX, DDB2, FDXR, TSPYL2 and p53 at days 2, 4 and 8 across low (0–1 Gy), medium (2–5 Gy) and high (6–10 Gy) dose ranges. Fold changes were computed by calculating the ratio of the post-exposure biomarker MFI to the mean pre-irradiation baseline MFI of control NHPs. The bars represent the mean, the error bars represent the standard error of mean (SEM), *p* value represent significance between dose groups by Welch’s ANOVA test.

For the NHP studies, larger blood volumes (1–2 mL) permitted the extension of the biomarker panel to include DDB2 and TSPYL2 which were identified as highly upregulated protein biomarkers following irradiation in our previous proteomic study^8^. Biomarkers ACTN1, BAX, DDB2, FDXR, TSPYL2 and p53 in NHP samples were tested over a wider dose range up to 10 Gy, on days 2, 4 and 8 after in vivo TBI exposure. Compared to the Hu-NSG study, fewer NHPs were irradiated and the NHP samples were divided into 3 dose groups: low dose (0–1 Gy; n = 25), medium dose (2–5 Gy; n = 8) and high dose (6–10 Gy; n = 11). Measurements of fold change indicate that biomarker expression levels are significantly higher at the medium to high dose range compared with the low dose (< 1 Gy) at day 2, day 4 and day 8 (the exact *p* values are marked in Fig. 4). In general, biomarker expression does not increase further after exposure to the high doses of radiation, except for DDB2 at day 8.

To assess the diagnostic ability of the biomarkers for radiological triage^33^, we performed ROC curve analysis to discriminate radiation doses below 2 Gy from doses equal to or above 2 Gy (Supplementary Figure S2 and Table 2). The results show that all biomarkers individually (except for p53 at day 8), were able to discriminate these two groups with AUCs ranging from 0.741 (FDXR at day 2) to 0.996 (ACTN1 at day 2). Furthermore, biomarkers BAX, p53, DDB2 and TSPYL2 performed better at the earlier time points (day 2 or day 4) while FDXR provided a better diagnostic power at the later time point (Day 8). Of note, ACTN1 provided the best discriminating potential to identify doses below 2 Gy and doses equal to or above 2 Gy at all three time points.

**Table 2:** Area under the ROC curve (AUC) values from six individual biomarkers and a combination of all six biomarkers to discriminate radiation dose below 2 Gy and doses equal to or above 2 Gy.

Day	Biomarker						
	ACTN1	BAX	FDXR	p53	DDB2	TSPYL2	Combination
Day 2	0.996 (*p* < 0.001)	0.988 (*p* < 0.001)	0.741 (*p* = 0.006)	0.873 (*p* < 0.001)	0.973 (*p* < 0.001)	0.977 (*p* < 0.001)	0.987 (*p* < 0.001)
Day 4	0.984 (*p* < 0.001)	0.9767 (*p* < 0.001)	0.765 (*p* = 0.017)	0.820 (*p* = 0.003)	0.744 (*p* = 0.008)	0.992 (*p* < 0.001)	0.976 (*p* < 0.001)
Day 8	0.979 (*p* < 0.001)	0.833 (*p* = 0.001)	0.833 (*p* = 0.002)	0.602 (*p* = 0.264)	0.863 (*p* < 0.001)	0.884 (*p* < 0.001)	0.986 (*p* < 0.001)

All six biomarkers were combined by first fitting the data to a multiple linear regression model (Supplementary Table S3) followed by ROC curve analysis. The calculated AUCs using the model fitted values across data points was > 0.97 (Supplementary Figure S2 and Table 2), indicating excellent discriminating power to identify doses equal to or above 2 Gy.

## Discussion

In the event of a radiological catastrophe, immediate triage would be accomplished through a combination of physical dosimetry, history of an individual’s location, clinical signs and symptoms, and individual hematology assessment, along with other biodosimetry methods^34^. The development of FDA-approved biodosimeters for early population triage has the potential to save hundreds of thousands of lives following a mass-casualty nuclear disaster. As far as we are aware, no biodosimetry methods have been approved by the U.S. Food and Drug Administration (FDA)^35^. Towards FDA approval, we have developed the FAST-DOSE assay system, a simple protein biomarker-based triage assay for the rapid immune-detection of radiation-responsive proteins in small volume blood samples.

One of the challenges for biodosimetry studies is that there is no perfect model for biomarker response indicative of human response days after radiation exposure, thus limiting the development and validation of biodosimeters. To date, the development and validation of radiation responsive biomarkers in vivo has relied heavily on rodent, minipig and more recently NHP models^23,36–40^. In the present work, we used two animal models, Hu-NSG mouse and NHP to support our FAST-DOSE biodosimetry device to estimate delivered dose using peripheral blood samples for early triage decisions within 8 days after radiation exposure.

Previously, we used Hu-NSG mice as an alternative model to validate human radiation biodosimetry standards and used the human hematopoietic system to investigate candidate protein marker expression in vivo after radiation exposure^8,34^. In the present study, we engrafted NSG mice with human fetal CD34+ stem cells to enrich the number of human cells in the mouse blood^41^. The advantage of this model is that the injected CD34+ cell population is enriched with CD38- stem cells leading to faster engraftment (12 weeks as opposed to 16 weeks for cord cells) and higher levels of sustained mature human cells in the mouse blood^42^. The results show that although human cell engraftment levels were similar to our earlier study^8^, the NSG mice engrafted with human fetal cells showed a larger percentage of surviving human leukocytes; approximately 50% as opposed to 25% using the human cord blood stem cell model measured 3 days after 1 Gy X-ray exposure. This allowed for the use of CD45+ cells from the peripheral blood of the Hu-NSG mice mouse as opposed to the mouse spleen in previous study^8^.

Table 1 and Supplementary Figure S1 show the performance of the highest-ranking protein panel to estimate delivered dose in human peripheral blood leukocytes. Doses 1 Gy and 3 Gy were chosen to represent a relatively low and high dose respectively, given that the LD_50/30_ of Hu-NSG mice is approximately 3–4 Gy^43^. At these doses, the biomarker data indicate that they were not only able to differ the dose above and below 2 Gy (Supplementary Table S1), but also to estimate the delivered dose (Supplementary Figure S1). The accuracy for dose estimation was between 0.04 to 0.61 Gy for biomarkers ACTN1, BAX, FDXR and phospho-p53. At the delivered dose of 3 Gy, the mean dose estimation was below 3 Gy across all 3 time points. This is likely due to the relatively high dose that the mice received whereby biomarker levels have begun to plateau. This was similarly observed for the highest doses used in the NHP model (Fig. 4). The limitation of the study was the small number of human stem cell donors (number = 3) and the reduced number of NHPs per data point. Thus, for some data points, the dose estimation is not within 0.5 Gy. Future studies with additional Hu-NSG mice, along with increasing the number of human stem cell donors may enhance the accuracy of dose estimation. Individually, the top-ranking biomarkers can be used for dose estimation with results showing that ACTN1 at day 1, p53 + BAX at day 2 and p53 at day 3 individually produced relatively good dose reconstruction. However, the fact that human blood samples will be collected from hundreds of thousands of individuals over a wide range of time points following radiological exposure, highlights the potential requirement for the use of a combination of biomarkers for dose reconstruction. This may also extend the window of time relevant for medical decision making for radiological triage^44^.

The FAST-DOSE biomarkers were further tested in peripheral blood samples collected from NHPs exposed to a large range of acute doses up to 8 days after TBI exposure. Compared to the Hu-NSG mouse study, fewer NHPs were irradiated which limited the statistical power for generating dose curves. By grouping NHPs into low (0–1 Gy), medium (2–5 Gy) and high doses (6–10 Gy), the time-dependent variation in biomarker expression for all 6 biomarkers was demonstrated. The results also indicated that ACTN1, BAX, FDXR, phospho-p53 and TSPYL2 biomarker levels plateaued at the highest doses, whereas DDB2 begins to show an apparent dose response at the higher dose range by day 8. Interestingly, ex vivo studies using human blood have also found that *DDB2* gene transcription level can estimate relatively higher doses (4 Gy) compared with other genes (*CCNG1, BBC3,* etc.) that are better suited to estimate low doses only (< 0.1 Gy)^11^. At the gene transcription level, all the biomarkers tested here have been shown to be radiation responsive markers in multiple tissue types^11,13,45,46^ while several of them (BAX, FDXR, DDB2 and p53) have been used in the development of a gene expression-based signature for the reconstruction of dose in human peripheral blood^45,47–50^. We consider that future studies should further investigate the gene to protein relationship of these blood biomarkers for radiation biodosimetry.

In the case of a radiological incident, the “classification threshold” is defined as a cut off for the consideration of urgent treatment. Emergency planning guidelines have proposed a threshold dose of 2 Gy, such that individuals exposed to doses of radiation above 2 Gy are at higher risk for experiencing acute radiation syndrome (ARS). Individuals exposed to doses greater than 2 Gy are more likely to develop life-threatening symptoms of radiation exposure such as hematopoietic ARS (H-ARS; 2–6 Gy) and gastrointestinal ARS (GI-ARS; > 6 Gy) and will therefore benefit most from prompt treatment with appropriate countermeasures^24,51^. Supplementary Figure S2 and Table 2 shows that biomarkers BAX, p53, DDB2 and TSPYL2 performed better at the earlier time points while FDXR provided better dose discrimination power at later time points. Furthermore, ACTN1 provided the best discriminating potential to identify doses below and above 2 Gy at all three time points. When all six biomarkers were combined for ROC curve analysis, the calculated AUCs across data points was > 0.97 across all the days. These results suggest that individually, or in combination, all biomarkers possess potential for use as biodosimeters for radiation triage.

There are several advantages of using IFC technology to quantify the FAST-DOSE protein biomarkers as part of the FAST-DOSE system. IFC permits high throughput cellular image capture, allowing visual confirmation and automated analysis of peripheral blood leukocytes in the IDEAS software. This approach is advantageous over visual microscopy methods which are time consuming, impractical for triage following a radiological emergency and lack the statistical power of higher throughput methods^7,9^. Furthermore, the ability to analyze and quantify the intracellular staining patterns of each biomarker on a cell-by-cells basis using IFC is advantageous over conventional flow cytometry methods^10^, where only whole population intensities can be quantified. In addition, the IDEAS software allows for label-free detection and elimination of apoptotic lymphocytes, which can interfere with biomarker expression, based on only BF cell morphology. The absence of introducing an apoptosis-specific marker to eliminate these events will increase the speed at which samples can be processed and will reduce the cost of performing these assays. Furthermore, the ability to create a common data analysis template that can be applied to all biomarkers increasing the speed with which results can be obtained and reduces the time to answer, a critical consideration following a radiological emergency. We acknowledge that our IFC-based assay system is generally not portable, we anticipate that in the future the FAST-DOSE biomarker(s) can be transitioned and validated for use in a point-of-care (POC) device.

In general, the radiosensitivity of cells is directly proportional to the rate of cell division and is inversely proportional to the degree of cell differentiation^26^. Thus, the hematopoietic system is more susceptible to radiation injury than other organ systems following total body irradiation^26,52^. Figure 1 demonstrated that both human T cells and B cells decreased from day 1 to day 3 following irradiation, with B cells being more sensitive to ionizing radiation, a response that has been demonstrated in other publications by Kachikwu et al. and Bogdandi et al.^53,54^. In the NHP study, leukocyte counts were measured on days 1, 3 and 7 after the irradiation (data not shown). As expected, the leukocyte counts significantly decreased with delivered dose at day 3 and day 7. Recently, Hu and colleagues applied their Hemodose model^55^ to simulate the temporal profiles of granulocyte, lymphocyte, and platelet counts from two cohorts of Chernobyl accident patients and showed that the depletion kinetics of lymphocytes is early and rapid; reaching the nadir within 1–2 days (5 Gy) and 3–4 days (1 Gy). Although, these findings highlight the importance of blood counts as sensitive biomarkers of radiation exposure, their use as an effective single parameter biodosimeter is limited by the fact that baseline blood cell levels are highly variable within the healthy population and can be highly affected by underlying disorders such as cancer, infection and trauma^56,57^.

Future studies are planned to develop a multicolor immunoassay approach for the simultaneous detection of specific FAST-DOSE protein biomarker(s) in combination with surface antigens for specific blood leukocyte sub-types (T cell, B cell neutrophils etc.). The combination of the FAST-DOSE protein panel with hematological blood surface biomarkers also presents a promising approach to provide useful diagnostic information for the severity of hematopoietic ARS in the early days following radiation exposure, as well as potentially increasing the radiation-induced biomarker signal in the mixed-cell blood sample, permitting more accurate dose estimations^58^. As part of further development studies, a large population study should be considered to address biomarker sensitivity to accurately reconstruct absorbed dose. The development of mathematical models to estimate radiation dose based on measured biomarker levels that incorporate demographic and confounding factors such as age, race and sex will be required. The inclusion of potentially confounding populations whereby human subjects tested with various pre-existing medical conditions such as inflammation, trauma etc. could also further help identify the specificity of these biomarkers as well as their applicability limitations.

We also envisage that the FAST-DOSE assay could be adapted for use in the clinic to potentially aid radiotherapy treatment planning. The assay system can easily be configured for the detection of a variety of biomarkers within a complex biological sample and serves as a high-throughput platform for the transition of biomarker(s) and practical assays to the clinic and basic research. For instance, the FAST-DOSE protein panel includes biomarkers involved in DNA repair, apoptosis and senescence in human leukocytes. These endpoints may be useful early predictors of individual sensitivity to acute radiation injury during a variety of radiotherapy treatment regimens to monitor risk and biomarker response among patients which could permit adaptation to avoid negative outcomes from over- or under-dosing (e.g. toxicity or poor tumor control).

This study has presented protein biomarker-based FAST-DOSE biodosimetry system and has demonstrated its feasibility to estimate delivered dose from peripheral blood samples for early triage decisions within 8 days following radiation exposure. Our vision for future development is to construct a more simplified, faster FAST-DOSE assay system whereby biomarkers could be developed and transitioned for use within an FDA-approved, point-of-care (POC) device.

## Methods

### Animal model

Humanized mouse experiments were approved by the Institutional Animal Care and Use Committee (IACUC; approved protocol AAP9613) and were conducted under all relevant federal and state guidelines. Female immunodeficient NOD. Cg-*Prkdc*^*scid*^* Il2rg*^*tm1Wjl*^/SzJ (NSG) mice (The Jackson Laboratory; Bar Harbor, ME, USA), aged 6 to 8 weeks, were engrafted with commercially available human fetal CD34+ cells (n = 3 donors; 200,000 cells per mouse) from Advanced Bioscience Resource, Inc (Alameda, CA). Rhesus macaques (*Macaca mulatta*) consisting of an equal mix of males and females aged between 3 and 6 years and body weights ≥ 4 kg were obtained commercially from Worldwide Primates (Miami, FL) and housed at the Lovelace Biomedical and Environmental Research Institute (LBERI, Albuquerque, NM) under approved IACUC Protocol # FY18-106. All animal welfare procedures followed Public Health Service Policy on Humane Care and Use of Laboratory Animals, according to the Office of Laboratory Animal Welfare (OLAW), National Institutes of Health. These animals were part of a larger study designed to support the optimization of other biodosimetry system for use in a large-scale emergency radiation scenario^58^.

### Animal irradiation and blood sample collection

#### Humanized mice

Mice were successfully engrafted by 4–5 months and human cell engraftment was tested according to previous work^34^. X-ray irradiation was performed using X-RAD 320 biological irradiator (Precision X-Ray Inc., North Branford, CT) operated at 320 kVp, a current of 12.5 mA and dose rate of 0.88 Gy/min. For in vivo irradiations, mice were placed in a specifically designed mouse irradiation holder (Precision X-ray). Control mice were sham irradiated. All doses were validated using a Radcal ion chamber (Monrovia, CA) placed in the mouse holder. During the actual irradiations, the delivered dose was measured by placing the ion chamber at the same position into the mouse holder. A total of 46 humanized mice from 3 donors were randomly assigned to 3 irradiation groups 0 Gy (n = 14), 1 Gy (n = 16) and 3 Gy (n = 16). Blood samples were collected on days 1, 2 and 3 after irradiation. Peripheral whole blood samples were collected from each mouse by cardiac puncture using a heparin-coated syringe. Human leukocyte, T cell and B cell counts were determined by flow cytometry using 20 μL of heparinized blood following the standard flow cytometry (CytoFLEX, Beckman Coulter, Pasedena, CA) surface staining protocol as mentioned previously^5,34^. Blood cells were stained with the following human surface biomarkers: CD45, (white blood cell marker, clone HI30, Biolegend, San Diego, CA), CD3 (T cell marker, clone UCHT1; Biolegend), CD20 (B cell marker, clone 2H7; Biolegend). Analyses were performed using CytExpert Software (Beckman Coulter).

#### Non-human primates

Irradiations were conducted at LBERI using a research-dedicated 6 MV linear accelerator (LINAC; Clinac 600C, Varian Medical Systems, Palo Alto, CA), and calibrated to ± 2% absolute using a NIST-traceable PTW ionization chamber^58^. A detailed description of the irradiation procedure has been described previously^58^. Briefly, animals fasted overnight were sedated with 10 mg/kg (± 0.5 mg/kg) intramuscular ketamine and prior to irradiation were anesthetized with isoflurane (1–3% for maintenance, via face mask inhalation). The animals were exposed to total body photon irradiation (Day 0) except for the 0 Gy (sham) group who were also transferred to the LINAC facility and anesthetized but not irradiated. Irradiation consisted of the mid-plane target radiation dose delivered through a pair of right and left lateral opposed fields, each delivering one-half of the dose at a dose rate of 50–70 cGy/min depending on the individual animal dimension. A total of 44 NHPs were randomly grouped and irradiated up to 10 Gy (0 Gy, n = 23; 1 Gy, n = 2; 2 Gy, n = 2, 3 Gy, n = 2; 4 Gy, n = 2; 5 Gy, n = 2; 6 Gy, n = 3; 8 Gy, n = 4; 10 Gy, n = 4). In addition to standard care methods, the NHPs were administered daily oral antibiotics (Baytril, 5 mg/kg), Flintstones vitamins, and nutritional support (e.g. bananas, apples, oranges) after irradiation as well as fluid support (Prang), special diet (moistened biscuits), and anti-diarrheal as necessary^58^. Peripheral blood samples (1–2 mL) were collected in lithium-heparin vacutainer tubes (Becton-Dickson, Franklin Lakes, NJ) by venipuncture. Samples were collected from pre-irradiation as well as days 2, 4, and 8 post-irradiation and shipped at ambient temperature in insulated shippers (Pelican BioThermal, PN: Series 22-248), initially to a reference laboratory, aliquoted and then sent to FAST-DOSE assay. The total time from the blood draw to the assay was about 48 h.

### Imaging flow cytometry assay and analysis

Peripheral whole blood samples from humanized mice (300–600 µL) and NHPs (1–2 mL) were lysed with RBC lysis buffer (Thermo Fisher Scientific, Waltham, MA). Humanized mouse cells were first stained with rat anti-human CD45 (clone: YAML501.4, Thermo Fisher Scientific) for 30 min and then fixed using the FIX & PERM Cell Permeabilization Kit (Thermo Fisher Scientific), while NHP blood leukocytes were fixed without surface staining. Afterwards, cells were washed with perm/wash buffer from the kit and equally distributed to four 2D Matrix microtubes (Thermo Scientific, Waltham, MA) and stained intracellularly by one of the following rabbit polyclonal antibodies: ACTN1 (Cat: 3134S, Cell Signaling Technology, Danvers, MA), BAX (Cat: 5023S, Cell Signaling Technology), FDXR (Cat: HPA044393, Sigma, St Louis, MO), phosphor-p53 (p53) (Cat: 9289, Cell Signaling Technology), TSPYL2 (Cat:ab168860, Abcam, Cambridge, UK) and DDB2 (Cat: PA5-63568, Thermo Fisher Scientific) for 1 h at room temperature. Proper negative controls were included for intracellular staining (rabbit polyclonal IgG, Abcam). Secondary antibodies included, goat anti-rat Alexa Fluor 594 (A-11007, Invitrogen, for humanized mice only) and anti-rabbit Alexa Fluor 488 (A11034, Invitrogen, Waltham, MA). Samples were then washed with phosphate buffered saline (PBS), and measured using the ImageStream MkII Imaging Flow Cytometer (Luminex Corporation, Austin, TX) as described in our previous work^5–7^. For NHP cells, DRAQ5 nuclear dye (Life Technology, Carlsbad, CA) was added before measurement. Images of more than 5,000 cells per sample were acquired at 40× magnification using the 488 nm excitation laser. For compensation, cells stained with single fluorescence only were captured using the 488 nm laser with the brightfield and side scatter inactivated. The compensation coefficients were determined automatically by the compensation wizard and all captured images were analyzed within the IDEAS software (Luminex ver. 6.2.187).

### Statistical analysis

One or combination of several biomarker protein was used for reconstructing radiation doses applying linear regression. The capability of candidate biomarkers to estimate dose was compared using mean absolute error (MAE) of estimated dose vs. delivered dose. Overall model fit for sequential models was compared using Akaike Information Criterion with sample size correction (AICc), which accounts for both the statistical goodness of fit and the number of parameters required to achieve this particular degree of fit, by imposing a penalty for increasing the number of parameters. The lower the AICc score, the better the model is. Variance inflation factor (VIF) was used to quantify the severity of multicollinearity in the multiple linear regression model. Biomarker performance for classifying samples by dose categories below or above 2 Gy in the NHP blood samples was determined based on Receiver Operating Characteristic (ROC) curves, which allow characterization of the discrimination between two well-defined populations. Geometric mean fluorescence intensity (MFI) values were compared by Welch’s ANOVA test and Dunn’s post hoc test. Two-tailed *p* values less than 0.05 were considered statistically significant. Statistical analysis was performed using Microsoft Excel 2016 (Microsoft, Redmond, WA) and GraphPad Prism (version 6.01; GraphPad Software, Inc., La Jolla, CA).

## Supplementary information

Supplementary information.
