# Supplementary material for: Development of the FAST-DOSE assay system for high-throughput biodosimetry and radiation triage
Source: Sci Rep. 2020 Jul 29;10:12716. doi: 10.1038/s41598-020-69460-7 (PMC7392759; doi:10.1038/s41598-020-69460-7)
Supplement: Supplementary file 1 — Supplementary information. [file 41598_2020_69460_MOESM1_ESM.docx]

**Development of the FAST-DOSE assay system for high-throughput**

**biodosimetry and radiation triage**

Qi Wang^1,2*^, Younghyun Lee^1^, Igor Shuryak^1^, Monica Pujol Canadell^1^, Maria Taveras^1^, Jay R. Perrier^1,3^, Bezalel A. Bacon^1^, Matthew A. Rodrigues^4^, Richard Kowalski^3^, Christopher Capaccio^3^, David J. Brenner^1^, Helen C. Turner^1*^

^1^Center for Radiological Research, Columbia University Irving Medical Center, New York, NY 10032, USA

^2^ Radiation Oncology, Columbia University Irving Medical Center, New York, NY 10032, USA

^3^ ASELL, LLC, Owings Mills, MD 21117, USA

^4^ Luminex Corporation, Seattle, WA 98119, USA

* Corresponding Authors;

Qi Wang

Email: [qw2232@cumc.columbia.edu](mailto:qw2232@cumc.columbia.edu)

Helen C. Turner

Email: [ht2231@cumc.columbia.edu](mailto:ht2231@cumc.columbia.edu)

**Supplementary information**

**Supplementary Figure S1. Estimated dose (Gy) versus delivered dose (Gy) determined by FAST-DOSE biomarkers over 3 days post-irradiation.** Day 1 dose estimation is determined by ACTN1, day 2 dose estimation is determined by p53+BAX while day 3 dose estimation is p53. The solid black line represents values where the estimated dose equals the delivered dose and the small dashed and large dashed lines represent ±0.5 Gy and ±1 Gy, respectively. The error bars represent the standard error of mean (SEM) estimated dose.


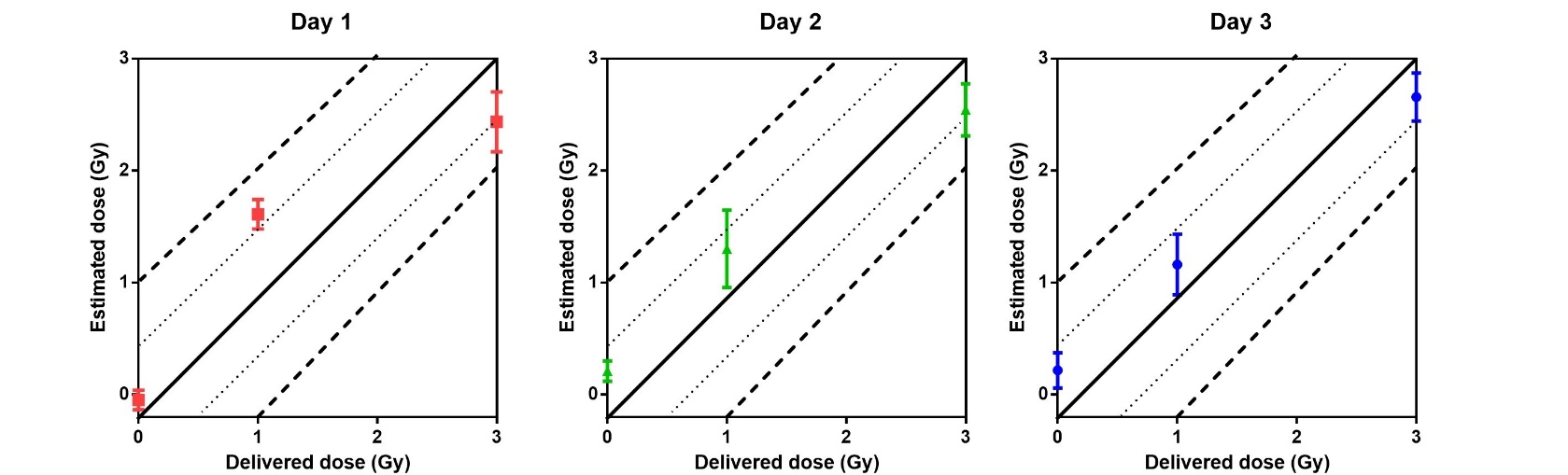


**Supplementary Figure S2.** **Receiver operating characteristic (ROC) curve analysis.** Receiver operation characteristic from six individual biomarkers as well as a combination of all six biomarkers to discriminate radiation dose below 2 Gy and doses equal to or above 2 Gy.


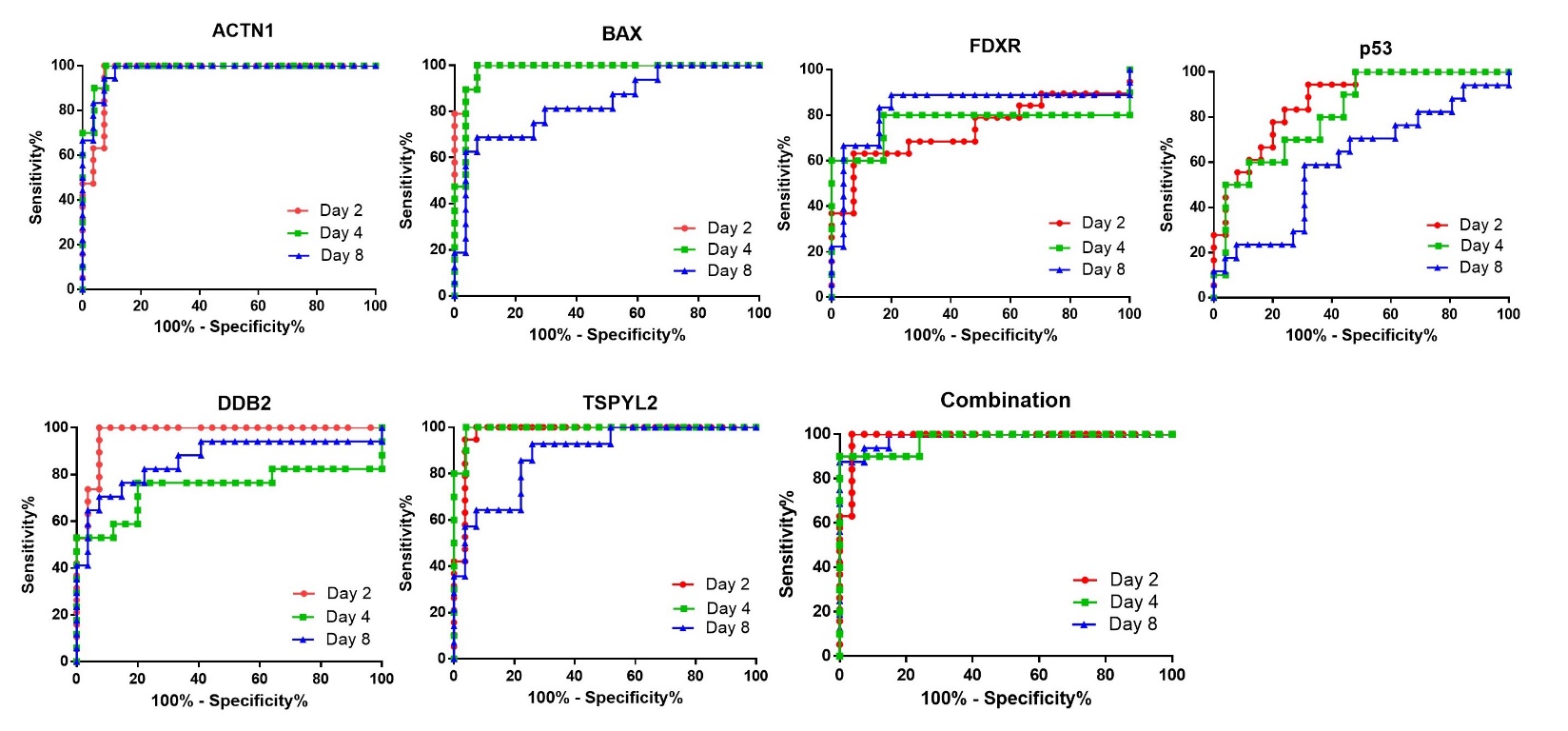


**Supplementary Table S1.** Area under the ROC curve (AUC) values from six individual biomarkers and a combination of all four biomarkers to discriminate low dose (0 Gy and 1 Gy) and high dose (3 Gy) in Hu-NSG model.

| **Biomarker**  **Day** | **ACTN1** | **BAX** | **FDXR** | **p53** |
| --- | --- | --- | --- | --- |
| Day 1 | 0.840 (p=0.037) | 0.840 (p=0.037) | 0.860 (p=0.027) | 0.880 (p=0.020) |
| Day 2 | 0.844 (p=0.039) | 0.933 (p=0.009) | 0.844 (p=0.038) | 0.911 (p=0.014) |
| Day 3 | 0.886 (p=0.028) | 0.667 (p=0.269) | 0.803 (p=0.044) | 0.985 (p<0.001) |

**Supplementary Table S2.** Variance inflation factor (VIF) from 4 individual biomarkers, combination of two biomarkers and combination of four biomarkers to discriminate low dose (0 Gy and 1 Gy) and high dose (3 Gy) in Hu-NSG model.

| **Day**  **Biomarkers** | **Day 1** | **Day 2** | **Day 3** |
| --- | --- | --- | --- |
| ACTN1 | 3.105 | 3.566 | 2.048 |
| BAX | 2.476 | 3.207 | 1.171 |
| FDXR | 3.074 | 3.322 | 1.517 |
| P53 | 3.633 | 2.008 | 2.729 |

**Supplementary Table S3.** Variance inflation factor (VIF) values were obtained by linear regression analysis from combination of 6 biomarkers in NHP model.

| **Day**  **Biomarkers** | **Day 2** | **Day 4** | **Day 8** |
| --- | --- | --- | --- |
| ACTN1 | 11.225 | 5.029 | 2.702 |
| BAX | 6.017 | 6.641 | 1.586 |
| FDXR | 2.356 | 2.213 | 3.557 |
| P53 | 5.387 | 1.977 | 1.507 |
| DDB2 | 7.137 | 4.693 | 1.716 |
| TSPYL2 | 6.285 | 5.168 | 1.711 |
